# Direct Numerical Simulations of Hotspot-induced Ignition in Homogeneous Hydrogen-air Pre-mixtures and Ignition Spot Tracking

**DOI:** 10.1007/s10494-017-9883-1

**Published:** 2018-01-08

**Authors:** Cheng Chi, Abouelmagd Abdelsamie, Dominique Thévenin

**Affiliations:** 1 Laboratory of Fluid Dynamics and Technical Flows, University of Magdeburg “Otto von Guericke”, Universitätsplatz 2, D-39106 Magdeburg, Germany; 2 Max Planck Institute for Dynamics of Complex Systems (Magdeburg), Magdeburg, Germany

**Keywords:** Hotspot ignition, Turbulence intensity, Direct numerical simulations, Misfire, Ignition spot tracking

## Abstract

A systematic study relying on Direct Numerical Simulations (DNS) of premixed hydrogen-air mixtures has been performed to investigate the hotspot ignition characteristics and ignition probability under turbulent conditions. An ignition diagram is first obtained under laminar conditions by a parametric study. The impact of turbulence intensity on ignition delays and ignition probability is then quantified in a statistically-significant manner by repeating a large number of independent DNS realizations. By tracking in a Lagrangian frame the ignition spot, the balance between heat diffusion and heat of chemical reaction is observed as function of time. The evolution of each chemical species and radicals at the ignition spot is checked and the mechanism leading to ignition or misfire are analyzed. It is observed that successful ignition is mostly connected to a sufficient build-up of a HO_2_ pool, ultimately initiating production of OH. Turbulence always delays ignition, and ignition probability goes to zero at sufficiently high turbulence intensity when keeping temperature and size of the initial hotspot constant.

## Introduction

Ignition is an important and complex issue, which has been extensively investigated during many decades. Early studies on ignition for safety analysis mostly relied on experimental measurements and simplified theoretical models, as reported by Lewis and von Elbe [1] (first published in 1951). Numerical studies are difficult since the underlying processes (chemical kinetics, turbulent transport, heat exchange) are fully coupled. It is very demanding to take into account all aspects simultaneously in the same numerical analysis. Hence, in the past, many authors have isolated one specific aspect and have concentrated their analysis on that point. Main challenges concerning chemistry are the poorly known chemical pathways and the large number of individual reactions for complex fuels. That is why the present study only considers stoichiometric hydrogen-air mixtures, for which accurate and validated physicochemical data are available [2]. Simulating turbulent flows is a challenge of its own. This is why the present study relies exclusively on Direct Numerical Simulations (DNS). Finally, the central challenge underlying heat exchange processes consists in modeling accurately all relevant paths (convection, diffusion, radiation). In order to obtain results independent from a specific geometry or confinement, only convection and diffusion are retained in the present study, radiative transfer in the gas phase being of minor importance for open-flow stoichiometric hydrogen-air mixtures.

Ignition was first studied for laminar, non-premixed flames [3, 4] using simple chemistry. Later, kinetic effects have been taken into account, e.g. [5–8]. Due to the dominating combustion mode at that time and to associated simplifications, ignition has been mostly investigated for non-premixed flames, as reviewed for instance in [9].

In the early nineties, numerical studies on ignition under turbulent conditions became possible due to the progress in computing power. A two-dimensional (2D) turbulent flow was considered in [10, 11] with simplified chemical kinetics. The results were discussed further in [12, 13], demonstrating in particular the interest of DNS to investigate such configurations. Further aspects, like composition inhomogeneities impact as well the auto-ignition process [14]. Later works considered more realistic kinetics in 2D flows (e.g., [15]) or looked at three-dimensional (3D) flows but with simplified kinetics (like in [16–18]). For example, Reddy et al. [19] conducted 2D numerical simulations of lean premixed natural gas-air combustion and studied the critical ignition energy and the influence of equivalence ratio, kernel temperature and size. It was found that as long as the available ignition energy is greater than a prescribed minimum value, the duration in which a steady flame speed is achieved is a strong function of kernel temperature; it is not a function of kernel size. A parametric study of auto-ignition scenarios for lean n-heptane/air [20] and hydrogen/air [21] mixtures with thermal stratification at constant volume and high pressure have been later conducted using 2D DNS, concentrating on the influence of imposed initial temperature fluctuations *T*^′^ and of the ratio of turbulence to ignition delay timescale. Further DNS studies indeed considered 3D flames with complex kinetic schemes (see e.g., [22]) but did not investigate specifically the ignition mechanism. The recent study in [23] investigated autoignition of DME/air turbulent mixtures, using a 2D parametric study and a single 3D case to check the possible effect of 3D turbulence. Zhou et al. [24] performed parametric 3D DNS with a skeletal chemistry for auto-ignition of a turbulent n-heptane spray. However, only 5 DNS cases have been actually considered, since a large number of 3D DNS realizations are impossible with current computer resources.

The present study is based on DNS with detailed chemical and transport models. A parametric investigation is carried out concerning ignition events induced by a hotspot in homogenous H_2_-air pre-mixtures at ambient temperature and pressure. An ignition diagram is first obtained to determine the critical combinations of hotspot temperature and radius leading to successful ignition or misfire. The same configurations are afterwards perturbed by adding turbulent fluctuations of increasing intensity. By repeating independent realizations for each turbulence level, the impact of turbulence on ignition probability and ignition delay is quantified in a statistically meaningful manner. To reveal the underlying mechanism controlling ignition, the ignition spot is identified and tracked in a Lagrangian frame. Unlike previous flame tracking studies [25–27], flame surface and flame structure are not of primary interest here, since a “flame” still does not exist as an established structure yet; thus, the tracking algorithm is governed only by fluid motion and turbulent fluctuations, and is used to compute the balance of the different physicochemical terms leading to ignition or misfire.

As 3D DNS involving accurate models for chemical, diffusion and thermodynamic processes are still not practical for systematic studies, the present work is mainly focused on 2D configurations, as done also for instance in [20, 21, 23, 28–30]. Yu et al. [31] compared 2D and 3D DNS of auto-ignition of a lean H_2_/air mixture, and concluded that 3D turbulence results in faster heat transfer rate than 2D. In the present study, several 3D cases have been computed as well in order to check the possible influence of 3D turbulence on ignition delay. Due to vortex stretching, which leads to a wider range of turbulent scales in 3D cases, the results might differ.

In Section 2, the problem configuration (including numerics, chemical kinetics, molecular transport, and initialization) and post-processing techniques are outlined, followed by a discussion of the numerical results in Section 3. Results from homogeneous laminar configurations are first presented (Section 3.1) to validate the employed numerical models with corresponding experimental data, and then obtain a laminar ignition diagram. The influence of turbulence intensity on the ignition probability and ignition delay is then discussed (Section 3.2). Finally, the findings obtained by tracking the hotspot in a Lagrangian frame are presented (Section 3.3), before concluding.

## Problem Configuration and Initialization

### Direct numerical simulations

DNS has emerged over the last decades as a tool providing an exact solution for both fluid dynamics and flame structures in reacting flows. As the computing power advances, DNS has become feasible for systematic study on ignition processes in 2D, providing accurate information on fluid dynamics and chemical composition. In the present study, the parallel DNS flame solver *DINO* [32] is used. It solves the low-Mach Navier-Stokes system coupled with detailed physicochemical models. The spatial derivatives are computed using a six-order centered explicit scheme. An explicit fourth-order Runge-Kutta time integrator is employed for temporal integration.

Chemical reactions are solved by Cantera [33] in *DINO*. For the results presented later, a H_2_/O_2_ chemical scheme suitable to investigate ignition phenomena [34] has been systematically employed. It involves 12 elementary reactions and 9 species (H_2_, O_2_, H_2_O, H, O, OH, HO_2_, H_2_*O*_2_, N_2_); reactions involving nitrogen are not considered.

As light radicals such as H or H_2_ play a noticeable role in hydrogen combustion [35], the thermo-diffusion effect (Soret effect) [36] is of significant importance and is always taken into account in the present DNS simulations. Full multicomponent diffusion may be numerically quite expensive [37]. Therefore, the Hirschfelder-Curtiss approximation [38] (also called mixture-averaged diffusion) is employed in the present study.

### Flame configurations and initialization

According to dimensionality, the initial flame configurations in this study are categorized into: 0D homogeneous pre-mixture with uniform temperature (referred hereafter as setup *S0D*); 1D homogeneous pre-mixture with a pulse in temperature (referred hereafter as setup *S1D*); 2D homogeneous pre-mixture with a circular hotspot at higher temperature (referred hereafter as setup *S2D*); and finally 3D homogeneous pre-mixture with a spherical hotspot at higher temperature (referred hereafter as setup *S3D*).

The first configuration *S0D* is used to validate the numerical and physicochemical models, specifically for premixed ignition studies. In this first instance, uniform initial profiles for all variables are imposed at *t* = 0 and allowed to iterate in a zero-dimensional simulation. In this way, the ignition delay *τ* is obtained as a function only of the mixture composition (described by the equivalence ratio Φ), and of the initial mixture temperature *T*_0_ and pressure *p*_0_.

**Fig. 1:**
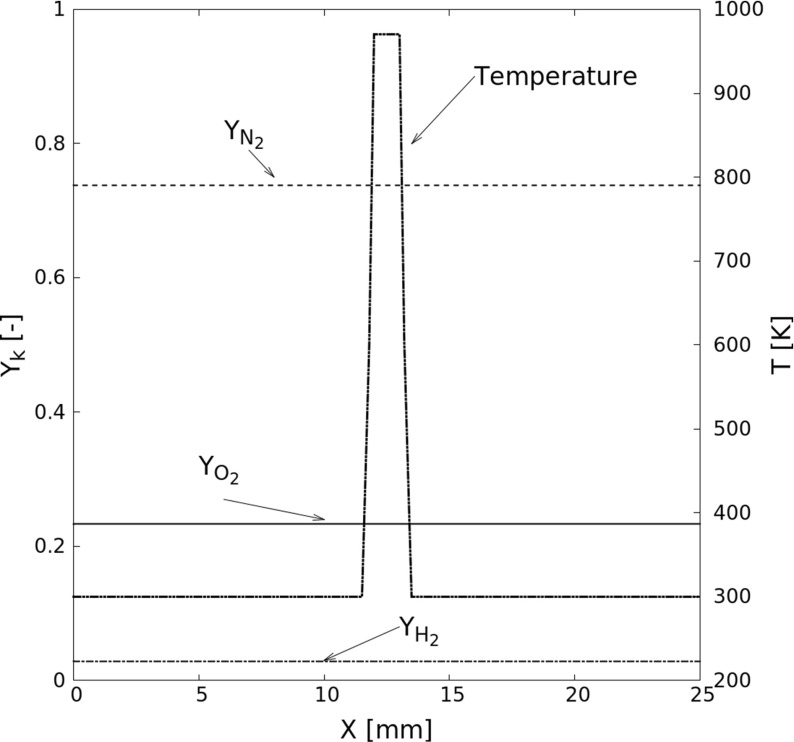
Typical computational domain and flame configuration showing *S1D* case – one-dimensional hotspot (initial configuration)

The second configuration *S1D* is illustrated in Fig. 1. Here, initial profiles are prescribed as *S0D* but in a one-dimensional configuration and this time with a pulse in temperature (and hence in density considering initially isobaric conditions at atmospheric pressure) in the middle of the domain. The third and fourth configurations *S2D* and *S3D* are similar to *S1D*, but in two and three dimensions respectively. In all *S1D*, *S2D* and *S3D* cases, the homogeneous pre-mixture is consisting of $Y_{H_{2}} = 0.029$, $Y_{O_{2}} = 0.233$ and $Y_{N_{2}} = 0.738$ (equivalence ratio Φ = 1 in air). The ignition induction time is investigated as a function of the initial temperature *T*_0_ within the hotspot and of the radius *R*_0_ of the initial hotspot (half-width of the initial hot temperature zone for *S1D*). The surrounding mixture has temperature *T*_*u*_ = 300 K. The step in temperature profile is approximated by a hyperbolic tangent function *ψ*, involving a stiffness parameter *𝜖* and the distance from the center *r*

1 $$\begin{array}{@{}rcl@{}} r &=& \sqrt{(x-0.5 \cdot L_{x})^{2}+(y-0.5 \cdot L_{y})^{2}+(z-0.5 \cdot L_{z})^{2}},\\ \psi &=& 0.5 \left( 1+{\tanh}\left( \frac{\epsilon \cdot (r-R_{0})}{R_{0}}\right)\right), \end{array} $$

where *𝜖* is the stiffness parameter, systematically set to 50 in this study. The parameters *L*_*x*_, *L*_*y*_ and *L*_*z*_ are the domain length in *x*, *y* and *z*-direction respectively. Preliminary studies have shown that the choice of the stiffness parameter leads to a negligible impact on the obtained results in the range considered, confirming observations of previous studies [39].

The domain is consisting of a line (for *S1D*), square (for *S2D*), cube (for *S3D*) of side length *L* = 1.6 cm discretized with a uniform grid (1024, 1024^2^ and 1024^3^ points, respectively), leading to a grid spacing of 15.625 *μ* m. Such a fine grid is necessary for DNS to resolve correctly stiff intermediate radicals like H_2_*O*_2_, and simultaneously the smallest vortical structures in the highest turbulence case in the present study (see later Table 1). The ”SuperMuc” supercomputer located in Munich (see https://www.lrz.de/services/compute/supermuc/systemdescription/ for more information) is used for all computations. For 2D cases, each run takes 30 minutes using 256 cpu cores; for 3D cases, each run takes almost five days using 1024 cpu cores in parallel. All the boundary conditions are periodic. Simulations are always stopped well before the reaction front reaches the boundary.

**Table 1:** Initial turbulence parameters for all simulated cases

Cases	*u*^′^/*s*_*L*_	*l*_*t*_ (mm)	*τ*_*f*_ (ms)	Re_*t*_	Da	Ka	*η* (mm)
1	0.25	0.902	0.99	22.8	363.8	0.013	0.07
2	0.35	1.293	1.01	45.6	256.6	0.026	0.06
3	0.48	1.727	1.00	82.1	196.3	0.046	0.052
4	0.61	2.213	0.99	136.2	144.8	0.081	0.045
5	0.67	2.442	1.00	164.9	131.9	0.097	0.043
6	0.78	2.860	1.00	224.8	114.1	0.131	0.038
7	0.96	3.387	0.97	327.7	93.0	0.195	0.036
8	1.13	4.057	0.95	462	78.4	0.274	0.033
9	1.28	4.323	0.95	557.7	70.4	0.335	0.031
10	1.37	4.914	0.99	678.5	66.1	0.394	0.03
11	1.43	5.199	1.01	749.3	64.7	0.423	0.03
12	1.47	5.364	1.01	794.6	61.9	0.455	0.029
13	1.53	5.505	1.00	848.9	59.8	0.487	0.029

**Fig. 2:**
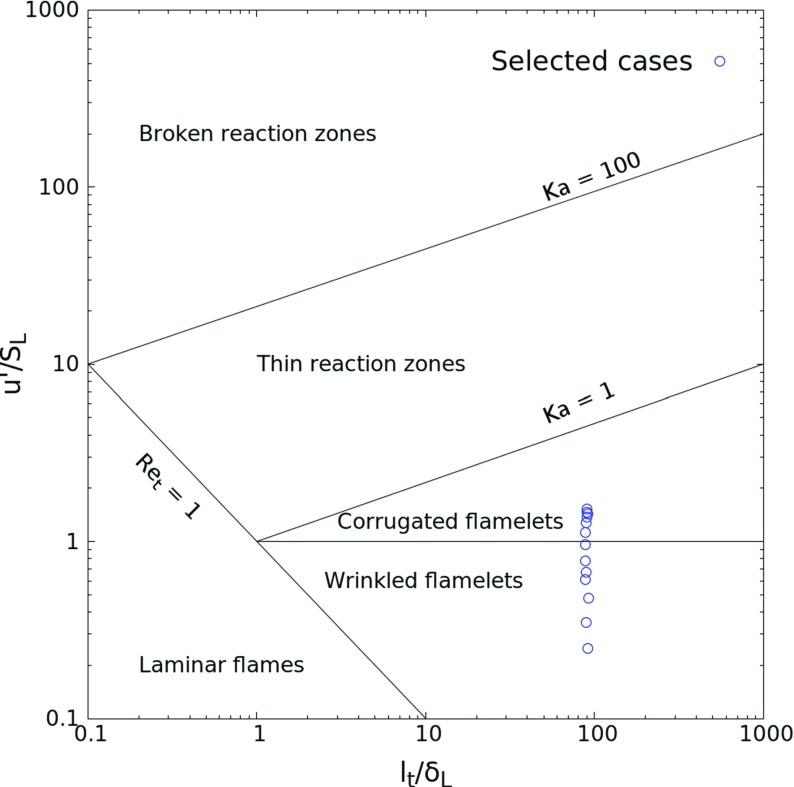
All the considered cases in the Borghi-Peters turbulent premixed combustion diagram [40]

For the turbulent cases, the initial laminar profiles are superimposed with a homogeneous isotropic turbulent field at *t* = 0, generated based on an Inverse Fast Fourier Transform (IFFT) with an analytically prescribed turbulence spectrum following von Kármán with Pao correction (VKP spectrum). Overall, 13 turbulence levels have been considered, ultimately spanning a wide range in turbulence intensities (0.25 ≤ *u*^′^/*s*_*L*_ ≤ 1.53) and integral-scale Reynolds number Re_*t*_ = *u*^′^*l*_*t*_/*ν* (22.8 ≤ Re_*t*_ ≤ 848.9), where *u*^′^, *s*_*L*_, *l*_*t*_ and *ν* are the root-mean-square (rms) of velocity fluctuations, laminar flame speed, integral length scale and mixture viscosity respectively. The laminar flame speed for the condition considered is *s*_*L*_ = 2.43 m/s. All turbulent cases are located in the turbulent premixed combustion diagram of [40] as shown in Fig. 2. The Damköhler number Da = *s*_*L*_^2^*l*_*t*_/*u*^′^*ν* and further information concerning all simulated cases are gathered in Table 1. In this table, *η* denotes the Kolmogorov length scale, *τ*_*f*_ is the characteristic time of turbulence, Ka is the Karlovitz number. It is observed that the cases lie in the wrinkled or corrugated flamelet regimes. However, the reader should keep in mind that the current project considers the ignition process, not an established flame.

To obtain statistically meaningful results, an average over a sufficient number of independent realizations is needed, as already discussed for instance in [41]. For this reason, fifteen realizations have been finally considered for those turbulence intensities. Since a random number generator is involved, each DNS is associated with the same global properties of turbulence (spectrum, correlations, fluctuations…) but corresponds to a different initial condition in space, and thus to a different realization. Finally, this study relies on 13×15 independent DNS runs in two dimensions, complemented by selected three-dimensional simulations. As shown in Table 2, the really obtained integral-scale Reynolds number Re_*t*_ for all those fifteen realizations of all cases have been checked, and show slight variations around the targeted values.

**Table 2:** Re_*t*_ for all runs of all simulated cases

Runs	C1	C2	C3	C4	C5	C6	C7	C8	C9	C10	C11	C12	C13
1	22.8	45.6	82.1	136.2	164.9	224.8	327.7	462.0	557.7	678.5	749.3	794.6	848.9
2	23.1	43.3	79.7	132.3	161.9	216.1	323.7	479.8	592.3	658.5	708.2	795.6	858.0
3	22.6	44.9	80.2	128.5	162.3	220.5	336.7	475.3	613.9	681.1	739.8	786.7	846.4
4	23.0	44.5	77.6	134.9	160.0	216.8	322.3	446.9	573.8	694.1	696.0	789.6	856.8
5	21.5	45.7	79.3	131.7	162.0	214.3	327.3	459.6	568.5	641.1	736.1	776.4	828.0
6	23.9	45.6	80.3	133.8	164.6	226.6	333.9	477.2	565.6	675.3	722.5	780.7	848.6
7	23.5	44.1	78.5	132.3	166.3	209.2	333.1	475.7	595.1	669.8	720.5	787.1	823.9
8	23.4	43.9	78.2	137.4	168.2	219.5	329.7	475.3	580.9	673.6	701.2	797.3	801.1
9	22.8	44.4	81.9	137.4	166.3	221.2	332.7	452.6	588.4	674.2	693.9	790.2	814.5
10	22.6	45.7	78.7	133.1	162.8	217.7	323.7	466.7	577.4	666.1	738.1	799.8	840.0
11	22.6	44.4	81.5	136.0	162.3	214.0	336.4	450.1	572.7	683.0	733.2	754.6	857.0
12	22.8	44.8	82.0	135.1	163.4	222.4	342.0	466.8	575.0	680.0	713.9	759.9	832.5
13	23.8	43.4	80.4	131.9	163.2	216.3	338.5	468.8	606.2	674.4	756.0	799.6	836.9
14	22.7	44.7	79.1	137.7	164.1	220.0	333.8	472.7	577.2	677.4	746.1	798.0	826.5
15	22.8	43.4	81.2	139.2	161.8	209.2	335.6	465.2	557.2	641.1	742.2	778.8	850.1

### Postprocessing and particle tracking

There are many criterias with which the auto- and/or hotspot-ignition delay, *τ*, of a flammable mixture can be defined: for instance based on the moment of fastest temperature rise, fastest gas expansion, fastest pressure rise, fastest reaction rate rise, fastest rise in a given species [42, 43]… All these definitions have convincing justifications depending on the particular focus of the study, although a disparity of up to 20% in the obtained delay times might be observed [39]. When considering detailed chemistry simulations, the ignition delay computed as the time of fastest temperature rise is often recommended for simple kinetic systems like hydrogen [39] and is used systematically in the present study. If ignition does not occur within 5 ms and the peak temperature is decreasing, the case is classified as misfire and the simulation is stopped.

In previous studies tracking a premixed flame (e.g., [26]), the position of a flame particle vs. time is typically described by,

2 $$ \frac{d\textbf{x}^{p}(t)}{dt} = \textbf{u}(\textbf{x}^{p}(t),t) + S_{d}(\textbf{x}^{p}(t),t) \textbf{n},  $$

where **x**^*p*^(*t*) is the flame particle position, **u**(**x**^*p*^(*t*),*t*) is the flow velocity at position **x**^*p*^(*t*), *S*_*d*_(**x**^*p*^(*t*),*t*) is the local displacement speed of the flame isosurface with **n** being the unit vector normal to the flame front.

However, in the present study, the “ignition spot” (and not the surface of a burning flame) is the target considered for tracking. This ignition spot is defined as the Lagrangian fluid particle that ignites first within the computational domain, in case of successful ignition. The local movement of this ignition spot does not correspond to the propagation of a flame front. Though a flame front would later develop, we are only interested in the initial (ignition) phase, before the establishment of any flame. Thus, instead of Eq. 2, tracking corresponds in the present case simply to,

3 $$ \frac{d\textbf{x}^{p}(t)}{dt} = \textbf{u}(\textbf{x}^{p}(t),t),  $$

as would be the case for a non-reacting flow. For the tracking algorithm, the initial position of the later ignition spot is required, but it is at first unknown. This is why the DNS simulations used for tracking purposes are repeated twice with exactly the same initial turbulence. The first DNS simulation delivers the overall ignition delay *τ* and the position at which ignition takes place. During this fist simulation, the Lagrangian pathlines of massless flow tracers placed at all grid points within the hot kernel or at a distance smaller than 0.5*R*_0_ from its initial boundary are tracked using standard tools implemented in DINO. Considering the large number of such points, only the starting position (at *t* = 0) and the current position (always overwritten) of the corresponding pathlines are kept in memory.

Using this approach, at the end of the first simulation, the initial position of the ignition spot (in a purely Lagrangian sense, Eq. 3) is known. Now, in the second DNS (with exactly the same field of turbulence), only the history of this specific point is tracked in time, now storing at each time-step of the DNS all parameters needed for the later analysis.

For cases that do not ignite (misfire), the initial candidate point which will lead to the highest temperature at the final simulation time is tracked using again the previous technique. Finally, both for cases leading to successful ignition or to misfire, all the relevant quantities (composition, temperature...) are stored along these trajectories in space and time for a later analysis.

## Numerical Results and Discussion

First, results in the laminar regime are discussed and an ignition chart involving critical ignition parameters in the absence of turbulence is derived. Then, by introducing turbulent fluctuations in the domain, the influence of turbulence intensity is discussed. Results are presented in a statistical manner towards characterizing successful ignition (or misfire) events relevant to safety issues. Finally, the underlying mechanism leading to successful ignition is analyzed by tracking the ignition spot in a Lagrangian frame.

### Ignition/misfire events under laminar conditions

**Fig. 3:**
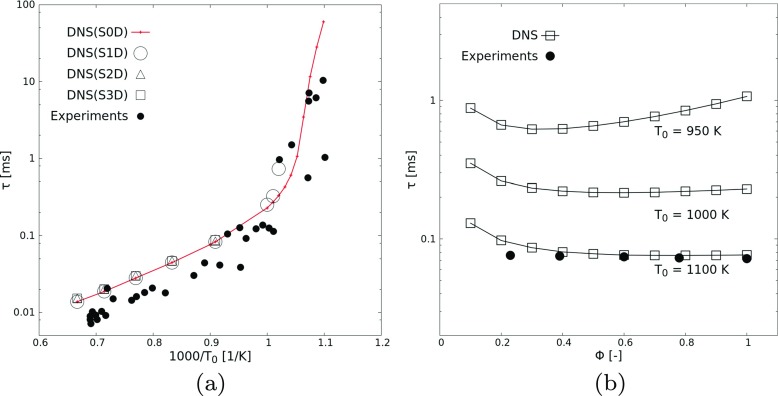
Ignition delay (*τ*) versus (**a**) initial temperature *T*_0_ for Φ = 1.0 (and *R*_0_ = 1 mm for setups *S1D, S2D, S3D*) and (**b**) mixture equivalence ratio (0.1 ≤ Φ ≤ 1.0 for setup *S0D*) of H_2_-air mixtures under laminar conditions, with corresponding experimental data from the literature [5, 45]

DNS in laminar conditions have been carried out for a wide range of initial hotspot temperatures (910 ≤ *T*_0_ ≤ 1500 K, for homogenous zero-dimensional cases *S0D* to three-dimensional spherical hotspots, *S3D*) and kernel radius (0.2 ≤ *R*_0_ ≤ 3.0 mm, for two-dimensional circular hotspots, *S2D*). In Fig. 3a, the ignition induction time *τ* is plotted as a function of *T*_0_. The induction times are found to decrease strongly with increasing initial mixture temperature. This result is expected in zero-dimensional cases since the ignition delay in such a configuration is governed only by the rate-limiting elementary reactions [44]. Strong competition between the chain branching and chain terminating reactions at higher temperatures leads to earlier ignition. With the other setups, a fixed half-width (*S1D*) or hotspot radius (*S2D,S3D*) of *R*_0_ = 1.0 mm is used. From Fig. 3a, it is obvious that all these cases show the same ignition delay at high temperatures. However, the critical temperature (denoted hereafter as $T_{0,c@R_{0}= 1 mm}$) for successful ignition is larger in *S2D* and in *S3D* than in *S1D* case; and that in *S1D* case is larger than for *S0D* case. The different critical temperature between *S0D* and *S1D* is due to heat diffusion to the cold surroundings in *S1D* case, which does not exist in *S0D* and obviously impacts noticeably ignition delay. The difference between *S1D*, *S2D*, and *S3D* cases is due to the associated change in the surface-to-volume ratio of the hot initial region. However, for sufficiently large values of *T*_0_ and *R*_0_, laminar ignition delays collapse together for all setups.

The variation of ignition delay with mixture equivalence ratio Φ for three initial temperatures (*T*_0_ = 950, 1000, and 1100 K) for setup *S0D* is shown in Fig. 3b. Unlike the strong dependence of *τ* on *T*_0_, there is no striking effect of Φ on *τ*, which is consistent with experimental findings [5]. At higher temperature (1100 K), ignition is very slightly faster for mixtures approaching stoichiometry from below. At lower temperatures (e.g., 950 K), the ignition delay first drops noticeably on the lean side before rising steadily till stoichiometry. As a whole, clear variations in ignition induction times across Φ are more visible at lower temperature values.

Experimental self-ignition delay data from the literatures [5, 45] for different *T*_0_ and Φ within the limits considered in the simulations have been included as well in Fig. 3 for setup *S0D*. Note that for the experimental data, different groups used different criteria to define ignition. Therefore, direct comparisons are more difficult. Nevertheless, this figure shows that the numerical predictions and experimental data are in good agreement over the whole range of considered conditions. Since the ignition delay is purely kinetically controlled in homogeneous systems, the suitability of the employed reaction mechanism is confirmed as well by this study.

**Fig. 4:**
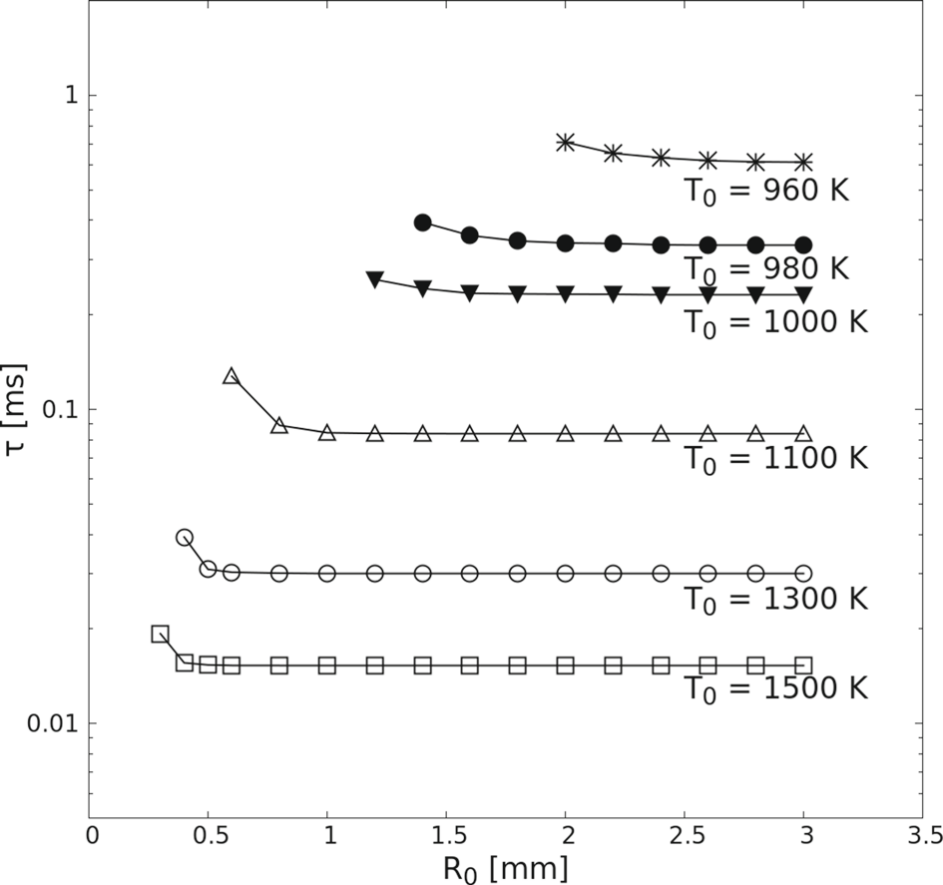
Hotspot ignition delay versus initial radius of hot kernel *R*_0_ for stoichiometric H_2_-air mixtures in setup *S2D*

In Fig. 4, the dependence of the ignition delay *τ* on the hotspot radius *R*_0_ for setup *S2D* is plotted. The minimum hotspot radius needed at a given hotspot temperature *T*_0_ to ignite the pre-mixture successfully is denoted here as the critical radius $R_{0,c@T_{0}}$. Starting from this critical radius, the hotspot-ignition delay first decreases rapidly with increasing initial size of the hotspot (via *R*_0_), before becoming constant for a sufficiently large value of *R*_0_. It is indeed expected that the ignition delay will not change when radius *R*_0_ is large enough, because at such condition the ignition delay is purely governed by the rate-limiting elementary reactions [44], leading to conditions similar to the zero-dimensional case. A hot region with a radius smaller than the critical radius *R*_0,*c*_ for a given *T*_0_ value does not lead eventually to a stable flame, since it does not harbor enough energy to trigger the chain reactions and compensate for heat loss to the surroundings. When radius *R*_0_ is larger than the critical radius $R_{0,c@T_{0}}$, the ignition delay is shorter for higher hotspot temperature. The relationship between ignition delay *τ* and hotspot temperature *T*_0_ at constant radius *R*_0_ is indirectly confirmed in Fig. 4.

**Fig. 5:**
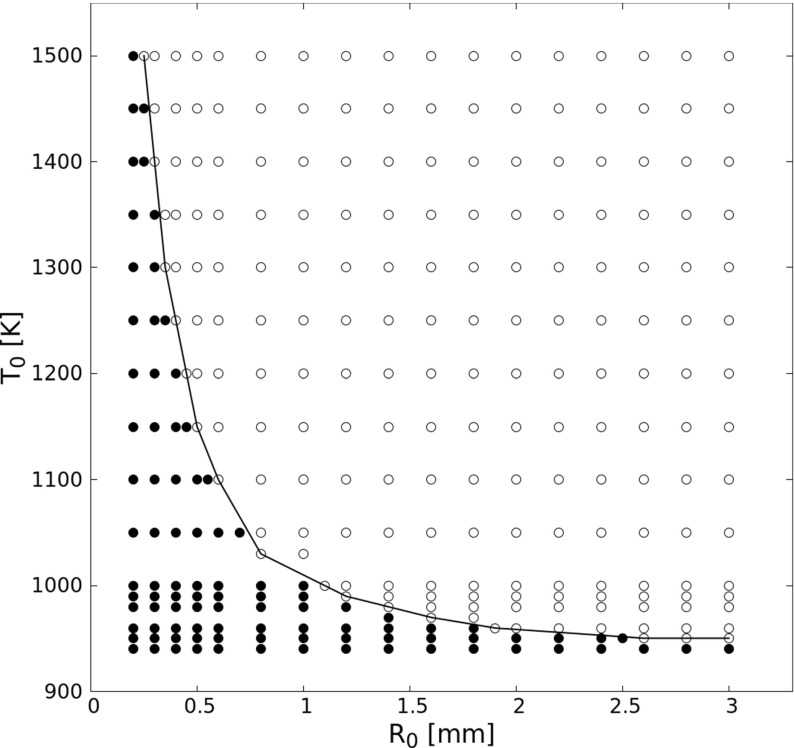
Hotspot-induced ignition diagram as a function of (*T*_0_,*R*_0_) for stoichiometric H_2_-air mixtures under laminar conditions in setup *S2D*. Successful ignition is shown with circle, misfire with dot

As discussed above, there is a critical temperature $T_{0,c@R_{0}}$ and a critical radius $R_{0,c@T_{0}}$ corresponding to the limit case for successful ignition. The entire spectrum of (*T*_0_, *R*_0_) combinations that lead to a successful ignition or misfired event for laminar, stoichiometric hydrogen-air pre-mixtures are plotted in Fig. 5. This ignition diagram shows successful ignition with circles and misfires with dots. A boundary curve separating ignition from misfire is shown in black. For this purpose, small variations in *R*_0_ down to 0.05 mm have been considered in order to obtain a better resolution around the ignition/misfire interface. Now combining Figs. 3 and 4, it is concluded that the ignition delay will decrease as the sample case moves from the boundary curve to the top side of the diagram and first rapidly decrease, then keep constant as the case moves from the boundary curve to the right side of the diagram.

For what follows, a single point (*T*_0_ = 1000 K, *R*_0_ = 1.2 mm) near the boundary curve at the ignition side of Fig. 5 has been chosen to check the impact of turbulence on ignition probability and ignition delay.

### Ignition or misfire events under turbulent conditions

Adding now time-decaying isotropic turbulence at various turbulence intensities, the influence of turbulence on the probability of hotspot-ignition events for premixed hydrogen-air mixtures can be quantified. As mentioned in the previous section, the hotspot is chosen with temperature *T*_0_ = 1000 K and radius *R*_0_ = 1.2 mm. This size is much larger than the Kolmogorov scale for all cases considered.

**Fig. 6:**
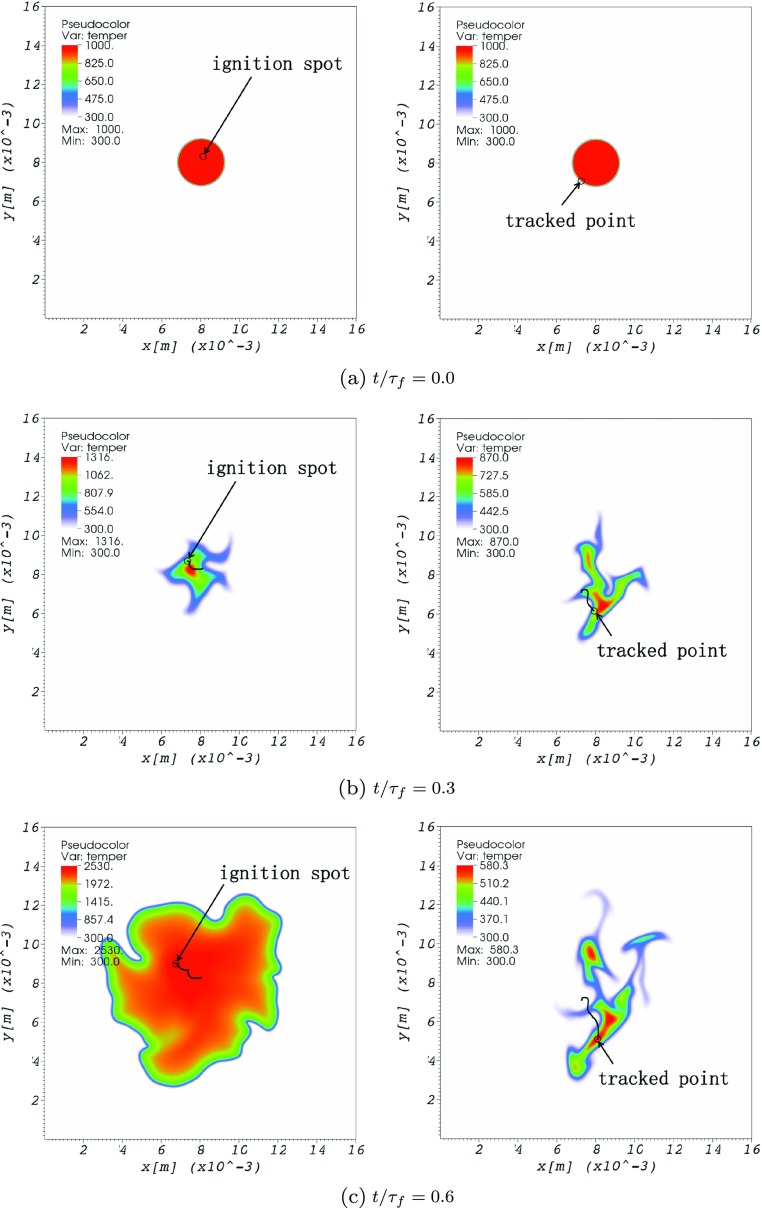
Exemplary temporal evolution of the temperature field during a successful (left column: *u*^′^/*s*_*L*_ = 0.78) and misfire (right column: *u*^′^/*s*_*L*_ = 1.13) event in a hotspot-induced ignition simulation of stoichiometric H_2_-air pre-mixtures under turbulent conditions for *T*_0_ = 1000 K and *R*_0_ = 1.2 mm. The color scale is always a min-max scale and is therefore different for each subfigure

The typical structure of the reaction zone is shown in Fig. 6, where the temperature fields are displayed at three different time instances for two selected realizations from Case 6 (*u*^′^/*s*_*L*_ = 0.78) and Case 8 (*u*^′^/*s*_*L*_ = 1.13), respectively. The impact of turbulence on the initially laminar kernel is very clear. In the first scenario (Fig. 6 - left column), the turbulence intensity is relatively mild. A smooth and quite homogeneous increase in heat release rate and consequently temperature is observed, resulting ultimately in a successful ignition event. In the second scenario (Fig. 6 - right column), it is a more hostile turbulent environment whose direct impact on the initial mixture leads to a misfire. Here, the turbulence intensity is considerably higher, resulting in enhanced mixing and diffusion of hot pockets within the surrounding cold mixture. The maximum temperature in the domain drops from 1000 K to well below 600 K with increasing time as the turbulence increasingly wrinkles the kernel. In this case, transport supersedes chemical reactions, ultimately leading to a complete quenching of the reactions. Remember that here, there are no initial temperature fluctuations in the hot region, which, if present, could have enabled the hot pockets to ignite earlier [46–48].

For a given turbulence intensity, each of the simulated turbulent cases has been finally repeated fifteen times, corresponding to independent realizations for a fixed value of *u*^′^/*s*_*L*_. The obtained ignition delays are listed in Table 3 for nine from the 13 cases presented in Table 1, selected to illustrate the typical trend in ignition delay under the influence of increasing turbulence stirring from left to right. Also included in Table 3 is the mean value of the ignition delay for the fifteen realizations. Note that this mean is obtained only from all successful ignition events. For these initial conditions, the corresponding 2D laminar case will always ignite at *τ*_0_ = 0.259 ms, which is hereby taken as a reference for comparisons.

**Table 3:** Hotspot ignition times (in ms) of atmospheric, stoichiometric H_2_-air mixtures with initially *T*_0_ = 1000 K, *R*_0_ = 1.2 mm for nine of the thirteen different turbulent conditions

Cases	1	3	4	6	8	9	10	11	13
Run 1	0.264	0.278	0.298	0.304	−	−	−	−	−
Run 2	0.268	0.273	0.298	0.343	0.354	−	−	−	−
Run 3	0.267	0.283	0.284	0.314	0.314	−	−	−	−
Run 4	0.267	0.323	0.279	0.293	−	−	−	−	−
Run 5	0.268	0.278	0.283	−	−	−	−	−	−
Run 6	0.267	0.279	0.302	0.279	0.342	−	−	−	−
Run 7	0.268	0.269	0.323	−	−	−	−	0.369	−
Run 8	0.263	0.273	−	0.289	−	−	−	−	−
Run 9	0.264	0.278	0.288	0.648	−	−	−	−	−
Run 10	0.264	0.283	0.303	0.287	−	−	−	−	−
Run 11	0.267	0.268	0.283	−	−	−	0.324	−	−
Run 12	0.269	0.268	0.299	0.278	0.313	−	−	−	−
Run 13	0.264	0.278	0.279	0.304	−	0.427	−	−	−
Run 14	0.264	0.278	0.334	−	−	−	−	−	−
Run 15	0.268	0.274	0.274	−	−	−	−	−	−
Average	0.266	0.279	0.295	0.334	0.331	0.427	0.324	0.369	−

Fifteen realizations are presented for each turbulence intensity. Entries with a hyphen (−) refer to a misfire event

**Fig. 7:**
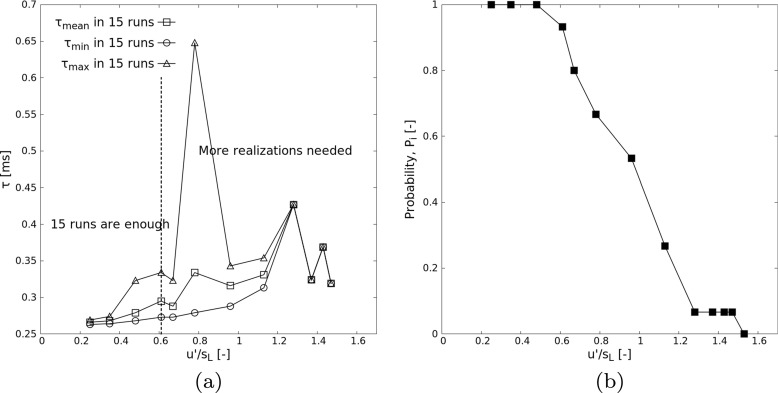
Hotspot-induced ignition: (**a**) ignition delay *τ*_*mean*_ and (**b**) ignition probability, *P*_*i*_ versus turbulence intensity (*u*^′^/*s*_*L*_) of atmospheric stoichiometric H_2_-air pre-mixtures computed for *T*_0_ = 1000 K, *R*_0_ = 1.2 mm

By checking the results in Table 3, the dependence of hotspot-induced ignition delay *τ* and ignition probability *P*_*i*_ on the increasing turbulence intensity is obtained as in Fig. 7. In these 15 realizations, when turbulence intensity is lower than 0.484 (corresponding to Re_*t*_ ≤ 82.1, Cases 1 to 3), all runs are leading to successful ignition. Increasing further turbulence intensity, ignition is not always observed any more; for Case 13 (corresponding to Re_*t*_ = 848.9), no ignition is observed at all. It is interesting to see that the ignition delay in all turbulent realizations is longer than the laminar ignition delay *τ*_0_ = 0.259 ms under same initial conditions (except turbulence), without exception. This confirms the school of thought that turbulence alone delays ignition of flammable mixtures compared to the corresponding case under laminar conditions, and never the other way round. However, this applies obviously only to cases with fully homogeneous initial conditions; the picture might be far more complicated when adding for instance inhomogeneities in dilution or equivalence ratio.

It is obvious from Fig. 7b that the probability of successful ignition is decreasing monotonically with the increasing turbulence intensity, from 1 (always ignites, for laminar conditions) to 0 (never ignites, at very high turbulence intensity). Starting first with Case 9, at most one successful ignition event happens in all 15 realizations; the ”mean” value for ignition delay is then a single value, and cannot be used for any statistics. At very high turbulence intensity, more realizations would be needed to obtain a statistical mean value of the ignition delay. This would also smooth out the probability curve. As shown in Fig. 7a, when *u*^′^/*s*_*L*_ > 0.61, the number of statistical samples becomes insufficient to calculate a proper mean ignition delay with a sufficient accuracy. Starting with Case 4, the mean ignition delay starts to oscillate around increasing values as the turbulence intensity increases. This is an indication that for Case 4 upwards, more realizations would be needed. Nonetheless, the monotonic increase of the ignition delay with turbulence intensity can still be guessed, and is clearer when looking at the shortest ignition time required in fifteen realizations, at least for those cases with *u*^′^/*s*_*L*_ ≤ 1.28.

**Fig. 8:**
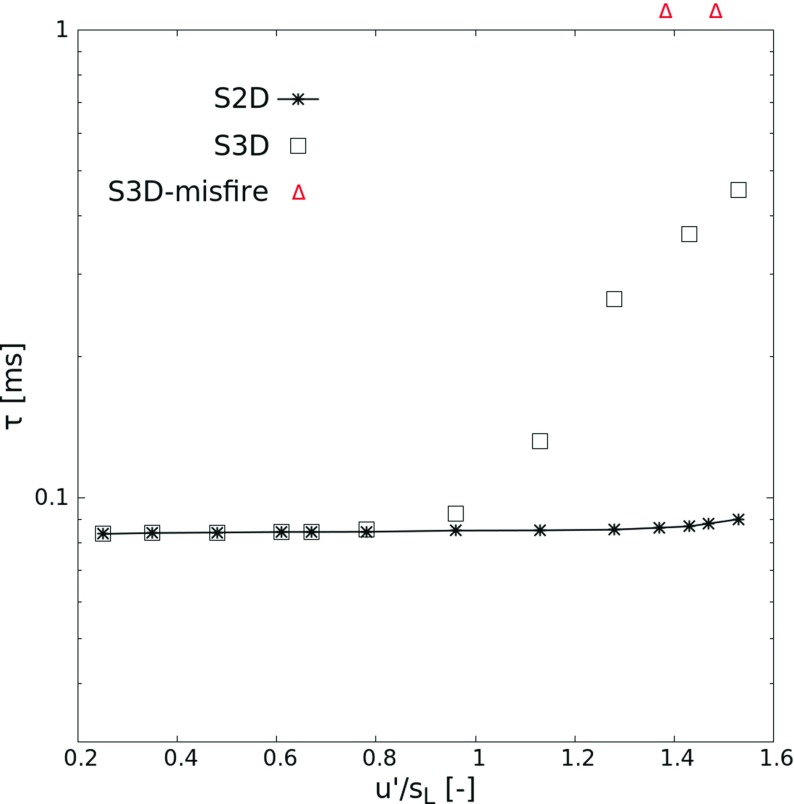
Hotspot ignition delay *τ* versus turbulence intensity (*u*^′^/*s*_*L*_) of atmospheric stoichiometric H_2_-air pre-mixtures computed for both 2D and 3D configurations. The hot kernel has an initial radius *R*_0_ = 1.2 mm and initial temperature *T*_0_ = 1100 K

Since these results have been obtained in 2D DNS, the possible impact of three-dimensional features should be checked once again. For this purpose, several 3D runs have been simulated with hotspot radius at *R*_0_ = 1.2 mm and *T*_0_ = 1100 K. This slightly higher temperature ensures that the 2D results deliver a nearly constant ignition delay for multiple realizations, even at high turbulence. For such conditions, the possible impact of three-dimensional features on ignition can be investigated separately from the effect of turbulence. As shown in Fig. 8, the ignition delay for S2D and S3D cases are completely identical at low turbulence intensity. This similarity between 2D and 3D is expected since the ignition delay *τ* is for these conditions much lower than the characteristic time scale of turbulence *τ*_*f*_, which means that the rate-limiting process is only chemical reaction; for such conditions, 3D turbulent features should show only little effect [49]. However, the same comparison reveals visible differences in the high-turbulence range, starting around Case 8. Under such conditions, the ignition delay in 3D is found to be systematically longer than that in 2D, and increases toward values close to *τ*_*f*_. This finding supports the idea that the mixing process becomes rate-limiting when the size of the large turbulent eddies (represented by the integral length scale *l*_*t*_) are sufficiently large [49]. In the present case, the corresponding critical ratio between integral length scale and hot spot diameter is *l*_*t*_/(2*R*_0_) ≈ 1.7. Vortex stretching in 3D strongly increases the scalar dissipation rate compared to 2D [49]. As will be shown in the next section, faster scalar dissipation rate results in longer time for HO_2_ to accumulate, leading finally to a longer ignition delay. Accordingly, successful ignition becomes less probable, as heat transfer rate is faster in 3D, dissipating the hotspot, as already discussed for instance in [31].

However, since a three-dimensional DNS takes roughly 1000 times as long as the same computation in 2D for the same number of computing cores, statistical studies involving a large number of realizations are still not feasible in 3D. Remembering that the main focus of this work is set on safety applications, this is not a major problem, since 2D DNS always deliver a conservative estimate, overestimating the rapidity and the probability of ignition at high turbulence intensity. Using predictions from 2D DNS, corresponding safety guidelines would be always on the safe side.

### Ignition point tracking and analysis

To figure out what controls successful ignition or misfire, the two runs corresponding to Case 6 and to Case 8 already shown in Fig. 6 have been analyzed further using Lagrangian tracking. The tracking trajectories have been plotted as black lines in Fig. 6.

**Fig. 9:**
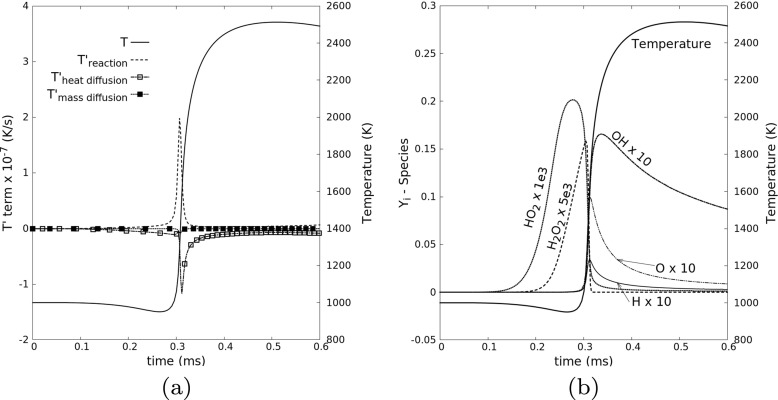
Temporal evolution of (**a**) the relevant contributions in the temperature transport equation and (**b**) the mass fractions of all intermediate species at the ignition point for one realization of Case 6, tracked in a Lagrangian frame, and leading to successful ignition

For Case 6, the ignition point is selected as the point with the highest heat release rate at ignition time. The contribution of heat diffusion, mass diffusion and reaction to the temperature rise in the transport equation for temperature are computed respectively using Einstein summation convention as

4 $$ T^{\prime}_{\text{heat diffusion}} = \frac{1}{\rho C_{p}} \frac{\partial}{\partial x_{j}}\left( \lambda \frac{\partial T}{\partial x_{j}}\right), $$

5 $$ T^{\prime}_{\text{mass diffusion}} = -\frac{1}{\rho C_{p}} \frac{\partial T}{\partial x_{j}} \sum\limits_{k = 1}^{N_{s}} \rho C_{p,k} Y_{k} V_{k,j}, $$

6 $$ T^{\prime}_{\text{reaction}} = -\frac{1}{\rho C_{p}} \sum\limits_{k = 1}^{N_{s}} h_{k} \dot{\omega}_{k}, $$

and are shown for the tracked point in Fig. 9a. Here, *C*_*p*_, *h*_*k*_, $ \dot {\omega }_{k}$, *λ*, *V*_*k*,*j*_ represent the specific heat capacity at constant pressure, specific enthalpy, mass reaction rate, heat diffusion coefficient and j-th component of the *k*-species molecular diffusion velocity, respectively. Variable *N*_*s*_ denotes the total number of species. The evolution in time of the mass fraction of all radicals and species have also been tracked for the ignition point, and are shown in Fig. 9b.

In Fig. 9a, a standard ignition picture is observed, as discussed in textbooks; temperature rises because of heat release produced by the chemical reactions, competing (successfully in this case) with heat diffusion as dominating sink term. The temperature change associated to mass diffusion is negligibly small in comparison to the other two contributions. By checking the evolution of each intermediate species in Fig. 9b, it is interesting to see radicals HO_2_, then H_2_*O*_2_, reach peaks just before the ignition starts, and radicals OH and O start to gain after HO_2_ and H_2_*O*_2_ are consumed. As is known, fast production of radical OH is the typical characteristics for ignition of hydrogen-air mixtures. It is confirmed that reactive radical OH is mostly generated by the chain branching reactions HO_2_ + H = OH + OH, and HO_2_ is produced by initial reaction H + O_2_ (+ M) = HO_2_ (+ M). The chain branching reactions H + O_2_ = OH + O and O + H_2_ = OH + H are not the major pathways in generating radical OH for the thermodynamic conditions in this study. This finding matches well with [43] and the recent work in [50]. As a conclusion, the local accumulation of radical HO_2_ is the real clue for successful ignition.

**Fig. 10:**
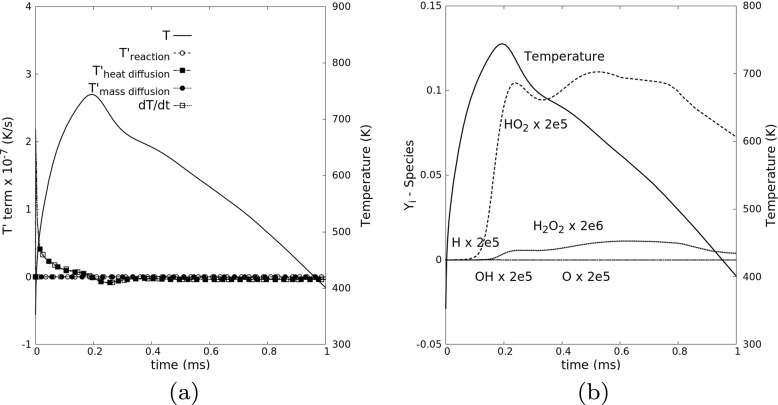
Temporal evolution of (**a**) the relevant contributions in the temperature transport equation and (**b**) the mass fractions of all intermediate species at the point that will reach highest temperature at the end of the simulation for one realization of Case 8, tracked in a Lagrangian frame, and leading to misfire

As seen from Fig. 9, the temperature at the ignition point does not drop too much from the hot initial temperature before ignition, due to the low level of heat diffusion $T^{\prime }_{\text {\scriptsize heat diffusion}}$ close to the kernel center under mild turbulent fluctuations. There are two reasons for the quick rise of HO_2_ in this case: (1) the temperature is high enough for fast initial reactions producing HO_2_; (2) the transport of HO_2_ aways from this region is slow, due to low turbulence. This statement can be confirmed by comparing with the results in the quenching case (Case 8), as shown in Fig. 10.

For the selected realization in Case 8, as already seen in Fig. 6 (right column), the point that will reach maximum temperature at the end of the simulation is not well within the kernel (as in Case 6), but at its boundary; hence, it is initially associated only to an intermediate temperature level, slightly above 350 K. The following temperature rise is largely dominated by heat diffusion from the hot kernel. After this rise in temperature, the radical HO_2_ starts to gain because of initial reaction, leading to peak temperature of almost 750 K, before dropping back. A noticeable production of OH is not observed. The small quantity of HO_2_ initially generated diffuses away due to intensive turbulent fluctuations. To summarize, there is not enough HO_2_ for starting the following chain-branching reactions for radical OH, and the mixture will not get ignited. Due to the very fast destruction of the hot kernel by intense turbulence, the conditions found within this kernel are even less favorable than at its boundary.

## Concluding Remarks

A systematic study has been done concerning the probability of successful hotspot-induced ignition of atmospheric H_2_-air pre-mixtures using Direct Numerical Simulations. Besides the slight influence of the mixture equivalence ratio, the ignition probability and ignition induction time are found to be mainly function of the initial hotspot temperature *T*_0_, hotspot radius *R*_0_, and turbulence intensity. An ignition diagram has been obtained for laminar conditions, allowing to determine regimes of successful ignition. Starting from the boundary curve in the ignition diagram, the ignition delay decreases as the initial kernel temperature *T*_0_ increases; the ignition delay first decreases, and then keep constant as the hotspot radius *R*_0_ increases. All the spherical kernels with initial (*T*_0_,*R*_0_) ≤ (*T*_0,*c*_,*R*_0,*c*_) will lead to misfire, even without any turbulence. The critical combination (*T*_0,*c*_,*R*_0,*c*_) is shown as a separation curve in the ignition diagram. With the increase of turbulence intensity, the probability of successful ignition is decreasing, the ignition induction time is becoming longer. For all turbulent realizations, the ignition delay is always larger than the laminar one, without exception.

The impact of three dimensional features has been checked by several 3D DNS realizations. The importance of the relative scale of ignition delay *τ* compared to the characteristic time scale of turbulence *τ*_*f*_ is confirmed. When the ignition delay *τ* is much lower than the characteristic time scale of turbulence *τ*_*f*_, corresponding here to a mild turbulent environment, the ignition delays of the 2D and 3D DNS are similar. At very high turbulence intensity, the ignition delay becomes longer in 3D DNS, and the ignition probability decreases due to stronger and faster dissipation.

The ignition point has been tracked in a Lagrangian frame to understand the ignition behavior, quantifying the trade-off between transport processes and chemical reactions. Initial reaction H + O_2_ (+ M) = HO_2_ (+ M) followed by chain-branching reaction HO_2_ + H = OH + OH are found to be critical for successful ignition at temperatures near 1000 K. When turbulence intensity is low, the mixing of the hotspot with cold surroundings is slow, the temperature around the ignition spot within the kernel remains high enough for the initial reaction to generate enough HO_2_. The diffusion of HO_2_ to the surroundings is also slow, which results in HO_2_ accumulation at the ignition spot. When HO_2_ reaches a certain level (typically a local mass fraction of the order of 10^− 4^ in this study), the chain-branching reaction producing OH becomes intensive, finally leading to successful ignition. When increasing turbulence intensity, the mixing becomes intensive, for both heat and active radicals such as HO_2_. Then, it becomes more difficult or even impossible to accumulate enough HO_2_ at any specific spot. The probability of successful ignition becomes lower. It may ignite for some realizations, but it will take more time for radicals HO_2_ to reach a sufficient level, leading to longer ignition times, and lowering the probability of a successful ignition.
